# IL-25 induces airway remodeling in asthma by orchestrating the phenotypic changes of epithelial cell and fibrocyte

**DOI:** 10.1186/s12931-023-02509-z

**Published:** 2023-08-27

**Authors:** Xiujuan Yao, Qinglin Chen, Xiangdong Wang, Xiaofang Liu, Luo Zhang

**Affiliations:** 1 Department of Respiratory and Critical Care Medicine, Beijing Tongren Hospital, Capital Medical University, No.2, Xinanhuan Road, Yizhuang District, Beijing, 100176 China; 2 Department of Otolaryngology Head and Neck Surgery, Beijing Tongren Hospital, Capital Medical University, Beijing, China; 3 Key Laboratory of Otolaryngology Head and Neck Surgery of Ministry of Education of China, Beijing Institute of Otolaryngology, No. 17, Hougou Hutong, Dongcheng District, Beijing, 100005 China

**Keywords:** Airway remodeling, Asthma, Bronchial epithelial cells, Fibrocytes, IL-25

## Abstract

**Background:**

Previous studies have shown that IL-25 levels are increased in patients with asthma with fixed airflow limitation (FAL). However, the mechanism by which IL-25 contributes to airway remodeling and FAL remains unclear. Here, we hypothesized that IL-25 facilitates pro-fibrotic phenotypic changes in bronchial epithelial cells (BECs) and circulating fibrocytes (CFs), orchestrates pathological crosstalk from BECs to CFs, and thereby contributes to airway remodeling and FAL.

**Methods:**

Fibrocytes from asthmatic patients with FAL and chronic asthma murine models were detected using flow cytometry, multiplex staining and multispectral imaging analysis. The effect of IL-25 on BECs and CFs and on the crosstalk between BECs and CFs was determined using cell culture and co-culture systems.

**Results:**

We found that asthmatic patients with FAL had higher numbers of IL-25 receptor (i.e., IL-17RB)^+^-CFs, which were negatively correlated with forced expiratory volume in 1 s/forced vital capacity (FEV_1_/FVC). The number of airway IL-17RB^+^-fibrocytes was significantly increased in ovalbumin (OVA)- and IL-25-induced asthmatic mice versus the control subjects. BECs stimulated with IL-25 exhibited an epithelial-mesenchymal transition (EMT)-like phenotypic changes. CFs stimulated with IL-25 produced high levels of extracellular matrix (ECM) proteins and connective tissue growth factors (CTGF). These profibrotic effects of IL-25 were partially blocked by the PI3K-AKT inhibitor LY294002. In the cell co-culture system, OVA-challenged BECs facilitated the migration and expression of ECM proteins and CTGF in CFs, which were markedly blocked using an anti-IL-17RB antibody.

**Conclusion:**

These results suggest that IL-25 may serve as a potential therapeutic target for asthmatic patients with FAL.

**Supplementary Information:**

The online version contains supplementary material available at 10.1186/s12931-023-02509-z.

## Introduction

Some patients with severe asthma demonstrate progressive and not fully reversible expiratory airflow obstruction despite the extensive use of inhaled corticosteroids (ICS), bronchodilators, and biologics [1, 2]. This specific phenotype can be called asthma with fixed airway obstruction (FAO), fixed airflow limitation (FAL), or chronic obstructive asthma (COA) [1, 3, 4]. FAL is common in patients with severe asthma [1, 3]. A national cross-sectional study showed that the prevalence of asthma with airflow limitation in China is 1.1%, representing 13.1 million Chinese adults [5].

The pathogenesis of FAL-associated asthma is largely unclear. The bronchial epithelium integrates complex inflammatory and remodeling processes in asthma by releasing IL-25, IL-33, and thymic stromal lymphopoietin (TSLP) [6–9]. Circulating fibrocytes (CFs) significantly accumulate in the peripheral circulation and asthmatic airway walls of patients with progressive, chronic obstructive, refractory, and severe asthma [10–14]. This indicates a possible link between bronchial epithelial cells (BECs) and CFs in orchestrating airway inflammation and remodeling via BEC-derived IL-25, IL-33, and TSLP.

In our previous study, we have shown that IL-25 and CFs could predict a distinct asthma phenotype in FAL [15]. In the present study, we hypothesized that the communication from BECs to CFs mediated by IL-25 contributes greatly to asthmatic airway remodeling and FAL. Because BECs also express the receptor for IL-25 [16], we hypothesized that IL-25 causes airway remodeling and FAL by promoting epithelial-mesenchymal transition (EMT)-like pro-fibrotic phenotypic changes in BECs in an autocrine manner. Experiments using clinical blood specimens from patients with asthma, an asthma murine model, and a cell culture/co-culture system were performed to prove these hypotheses.

## Methods

### Participants

This study included 80 adult patients with asthma who visited Beijing Tongren Hospital, Capital Medical University. Asthma was defined according to the Global Initiative for Asthma criteria (GINA, 2019; http://www.ginasthma.org). Patients who are active smokers or former smokers with a smoking history of ≥ 10 pack-years were excluded from this study. We also excluded asthma patients who were also diagnosed with chronic obstructive pulmonary disease (COPD) or with pulmonary imaging findings of emphysema. FAL is defined with a ratio of post-bronchodilator FEV_1_ to FVC (FEV_1_/FVC) of less than 0.70 [2, 17, 18].The demographic data of all patients were recorded. The study protocols were approved by the Institutional Research Ethics Committee of Beijing Tongren Hospital, Capital Medical University, and conducted in accordance with the Declaration of Helsinki. All subjects provided written informed consent before the conduct of the study.

### Flow cytometry

CFs in blood samples collected from patients with asthma during the first visit were identified using flow cytometry as previously described [13, 15, 19, 20].CFs were defined as CD45^+^ Collagen I^+^-PMBCs [21–23]. IL-25 receptor^+^-CFs were defined as IL-17RB^+^ CD45^+^Collagen I^+^-PMBCs [24–26]. The total CF count was expressed as the percentage of the whole population of PBMCs, while IL-17RB^+^-CFs were shown as a percentage of total CFs. The gating strategy is shown in Fig. S1. The detailed methods are provided in the supporting materials.

### Measurement of exhaled nitric oxide

The fraction of exhaled NO (FeNO) was measured using a portable NO analyzer (NIOX MINO, Aerocrine, Solna, Sweden) according to ATS/ERS recommendations [27].

### Murine model of asthma

Allergen- and non-allergen-driven mouse asthma models induced by ovalbumin (OVA) and IL-25, respectively, have been described in our previous studies [28–30]. All animal experiments were approved by the Institutional Research Ethics Committee of Beijing Tongren Hospital, Capital Medical University and performed in accordance with the guidelines of the Institutional Animal Care and Use Committee (IACUC).The detailed methods are presented in the Supporting Materials.

### Multiplex immunohistochemistry and multispectral image analysis

After euthanasia, the lungs of each mouse were removed and perfused with phosphate-buffered saline (PBS) via the heart to remove blood cells. The left lung was then inflated with 10% buffered formalin, embedded in paraffin, and sectioned at 4-μm thickness. Multiplex immunofluorescence staining and multispectral imaging were performed to identify the cell subsets co-expressing CD45, Collagen I, and IL-25R (IL-17RB) in the lung sections, as described previously [31]. CFs and IL-25 receptor^+^-CFs were identified as CD45^+^Collagen I^+^ and IL-17RB^+^ CD45^+^Collagen I^+^ cells, respectively. The detailed methods are provided in the supporting materials.

### Primary CFs culture and treatment

CFs were cultured as described previously [32–35]. CFs were then divided into four groups according to cell treatment, namely: (1) control group, (1%FBS-DMEM); (2) TGF-β1 treatment group (1%FBS-DMEM + TGF-β1[10 ng/ml, R&D Systems]); (3) IL-25 treatment group(1%FBS-DMEM + IL-25 [10 ng/ml, R&D Systems]); and (4) IL-25 + PI3K-antagonist group (1%FBS-DMEM + IL-25 [10 ng/ml] + LY294002 [15 μM, Sigma-Aldrich)]). Cells were then cultured for another 24 h and then harvested for further analyses, including real-time PCR, western blotting, ELISA, and cell proliferation and migration analyses.

### BECs culture and treatment

Sub-confluent BECs (Procell, Wuhan, HB, China) were serum-starved overnight and incubated with 1%FBS-RPMI 1640,1%FBS-RPMI 1640 + TGF-β1, 1%FBS- RPMI 1640 + IL-25, or 1%FBS- RPMI 1640 + IL-25 + LY294002, for 24 h. Cells were harvested for real-time PCR, western blotting, ELISA, cell migration, and epithelial-mesenchymal transition (EMT) analyses.

### Cell co-culture system

BECs were incubated with OVA (5, 10, and 100 μg/ml; Sigma-Aldrich)for 24 h to investigate whether BECs could respond to OVA by upregulating the expression of IL-25. In the co-culture system, BECs were suspended in RPMI 1640 medium at a density 1 × 10^5^ cells/ml and added into the upper transwell inserts (0.4-mm pore size;Thermo Fisher Scientific), while CFs were suspended in DMEM at a density 1 × 10^5^ cells/ml in the lower chamber pre-coated with 10 μg/mL fibronectin. Cells were then divided into three groups: (1) control group: BECs were incubated with 1% FBS- RPMI 1640 medium, and CFs were incubated with 1%FBS-DMEM; (2) OVA-treated group: BECs were incubated with 1% FBS- RPMI 1640 medium + OVA (10 μg/ml), while CFs were incubated with 1%FBS-DMEM; and (3) OVA + IL-17RB antibody-treated group: BECs were incubated with 1% FBS-RPMI 1640 medium + OVA (10 μg/ml), while CFs were incubated with 1%FBS-DMEM + rabbit anti-human IL-17RB polyclonal antibody (1 μg/ml, Proteintech, Rosemont, IL, USA). BECs and CFs were co-cultured for 24 h at 37 °C. Finally, CFs were harvested for real-time PCR, western blotting, ELISA, and cell migration analysis.

### ELISA

ELISA was performed to detect the concentrations of Collagen I, Collagen III, Fibronectin, connective tissue growth factor (CTGF), and IL-25 in culture or co-culture supernatants using commercial ELISA kits (R&D Systems, Inc), according to the manufacturers’ protocols. Data were read using the Infinite^®^ 200 PRO microplate reader (Tecan Group, Ltd.,Männedorf, Switzerland).

### Real-time RT-PCR

The mRNA levels of Collagen I, Collagen III, Fibronectin, CTGF, IL-25, and smooth muscle actin-α (α-SMA) in BECs or CFs were determined using real-time RT-PCR. The specific PCR primers are listed in Table S1. The detailed methods are provided in the supporting materials.

### Western blot analysis

Western blot analysis was performed with standard procedures (see the supporting materials).

### Cell growth and proliferation assays

Cell growth and proliferation were assessed using a Cell Counting Kit-8 assay (CCK-8, Abcam) according to the manufacturer’s instructions. CFs were seeded at a density of 1 × 10^3^ cells/ml in 96-well plates and incubated with 1%FBS-DMEM, 1%FBS-DMEM + TGF-β1, 1%FBS-DMEM + IL-25 or 1%FBS-DMEM + IL-25 + LY294002, as described above. Cells were then cultured for 24 h, 48 h, and 72 h in 5% CO_2_ incubator at 37 °C. At the end of the treatment, the CCK-8 solution was added directly to the test wells, and the plates were further incubated at 37 °C for 4 h. Absorbance at 450 nm was measured using the Infinite^®^ 200 PRO microplate reader (Tecan Group, Ltd.).

### Transwell migration assay

Treated BECs or CFs were seeded at a cell density of 1 × 10^6^ cells/ml into a 4 μm-pore membrane insert (Thermo Fisher Scientific) and placed into 10%FBS-RPMI 1640 and 10%FBS-DMEM, respectively. After incubating for 24 h at 37 °C with 5% CO_2_, cells underneath the insert were fixed with 4% formaldehyde for 30 min. The cells were then stained with 0.1% crystal violet for 5 min. The number of migrated BECs or CFs was visualized and assessed under a light microscope in three randomly selected fields per chamber.

### Statistical analysis

Statistical analyses were performed using the GraphPad Prism 8 software (GraphPad Software, San Diego, CA, USA). Parametric variables are expressed as the mean ± SEM, while nonparametric variables are reported as median [25th − 75th percentile]. Categorical variables are reported as numbers. For normally distributed data, the Student’s t-test was used for comparisons between two groups, while ANOVA followed by Tukey’s multiple-comparison test was used for comparisons between multiple groups. For data that were not normally distributed, the Mann-Whitney U test was used for comparison between two groups, while the Kruskal-Wallis test followed by Dunn’s multiple comparison test was used for more than two groups. The chi-squared test or Fisher’s exact test was used to analyze categorical variables. Spearman’s rank correlation coefficient was used to analyze bivariate correlations. A *P* value of < 0.05 was considered as statistically significant.

## Results

### Patient demographics

Eighty asthma patients were included in this study, among which 37 patients were included in the FAL classification (FEV_1_/FVC < 0.70). The clinical, demographic, physiological, and biological characteristics of the patients are presented in Table S2. Asthma patients with FAL are older and male dominant.

### Asthmatic patients with FAL had increased numbers of total and IL-17RB^+^-CFs

**Fig. 1:**
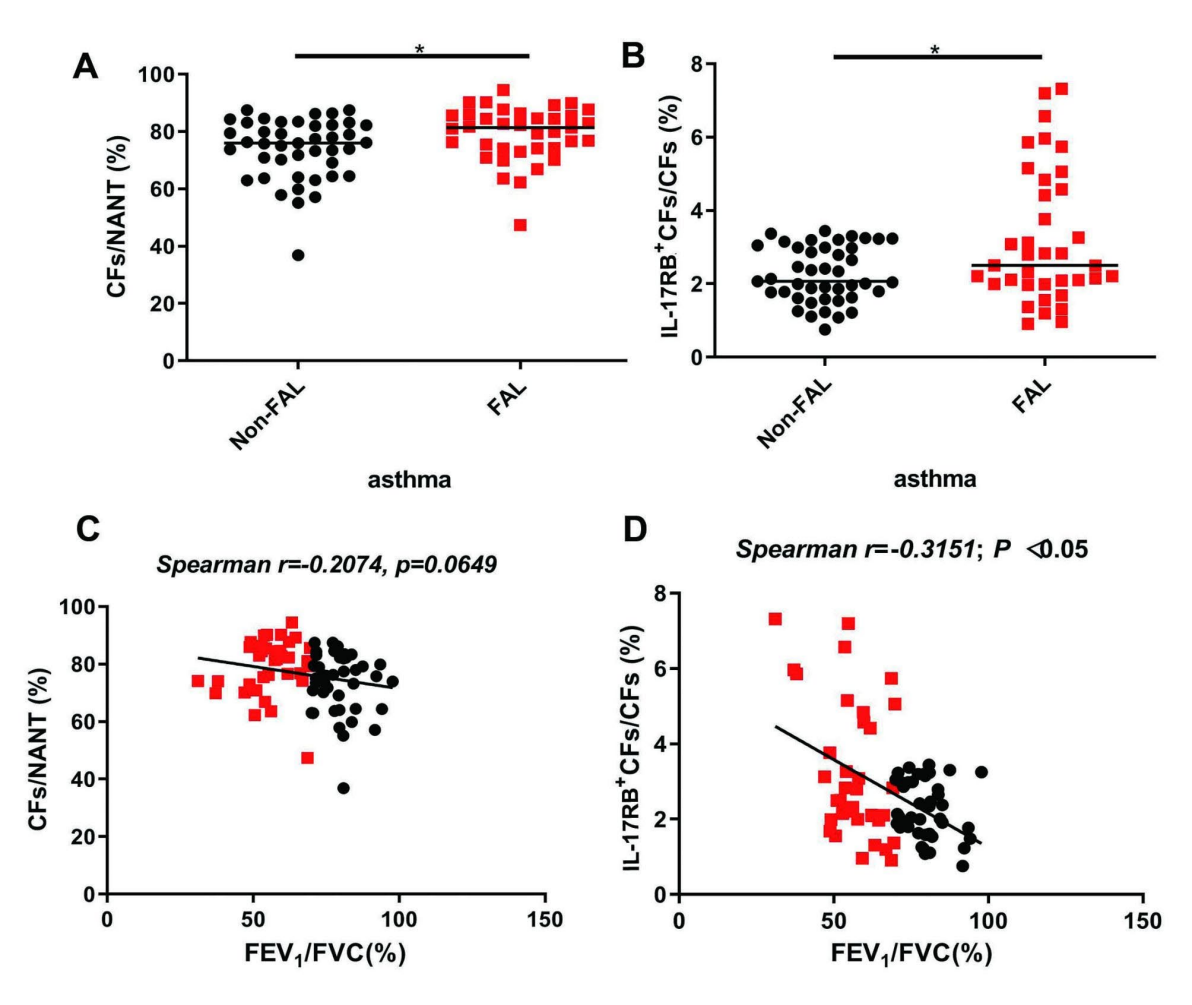
Increased total and IL-17RB^+^ circulating fibrocytes in asthma patients with FAL. **A and B**: CFs expressed as a percentage of NANT PBMCs (**A**) and and IL-17RB^+^-CFs expressed as a percentage of CFs (**B**) from asthma patients with and without FAL. Horizontal lines represent median. *p < 0.05, nonparametric *Mann-Whitney* test, *n* = 43 of patients without FAL and *n* = 37 of patients with FAL. **C and D**: Relationships between FEV_1_ /FCV and the percentage of total (**C**) and IL-17RB^+^ CFs (**D**) in patients with asthma were obtained by using nonparametric *Spearman* analysis

The percentages of total and IL-17RB^+^CFs in patients with asthma were significantly higher in the FAL group compared with those without FAL (Fig. 1A-B). A significant negative correlation between IL-17RB^+^CFs (but not total CFs) and FEV_1_/FVC was observed in patients with asthma (Fig. 1C-D),

### Total and IL-17RB ^+^-fibrocytes were increased in the lung tissues of experimental mice challenged with OVA and IL-25

**Fig. 2:**
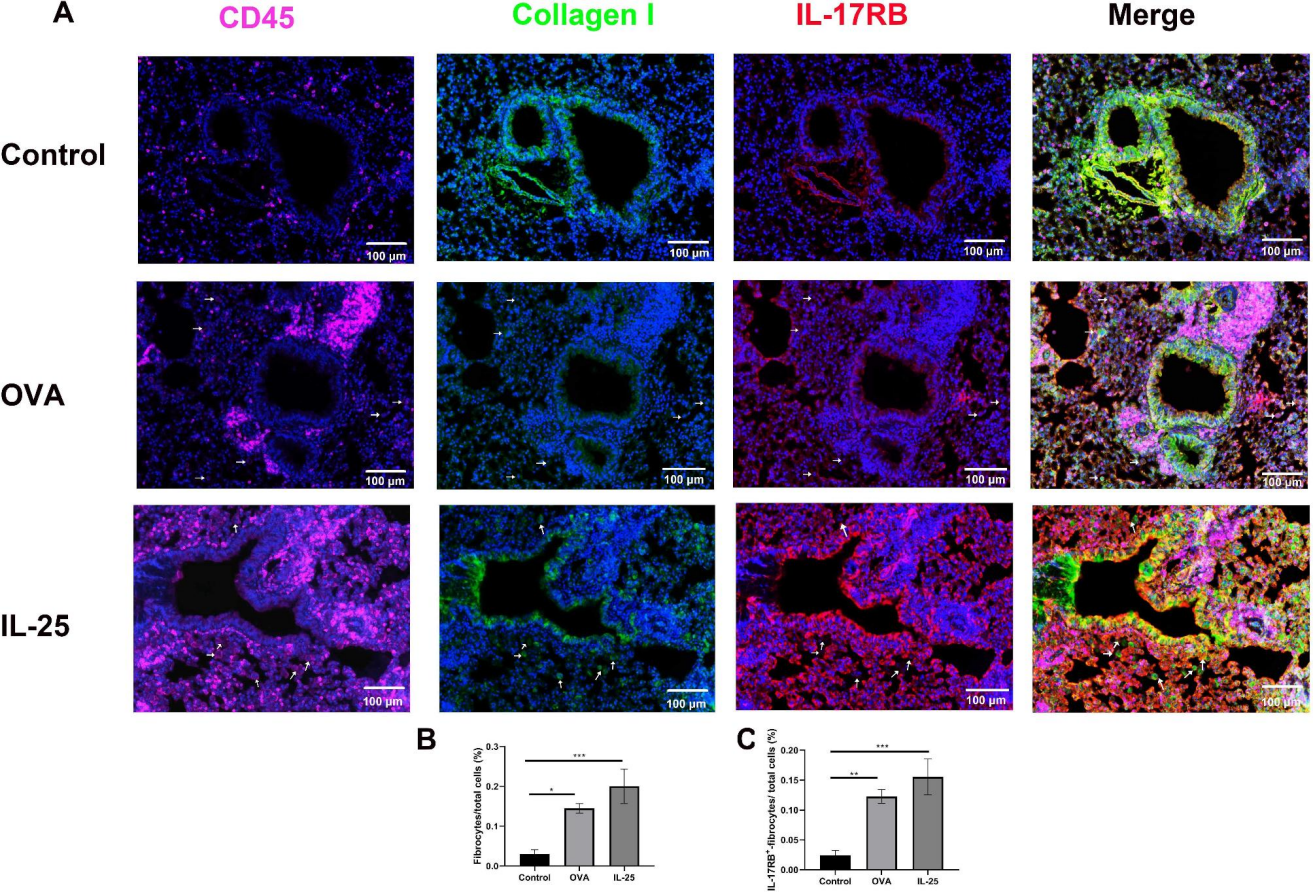
IL-17RB ^+^-CFs was increased in lung tissues of chronic mice asthma model. Multiplex IHC assay was used to determine the IL-17RB^+^-CFs in lung tissues. **A**: Representative images of multiplex IHC staining for the total fibroctyes (CD45^+^Collagen I^+^) and IL-17RB expressing fibrocytes (IL-17RB^+^ CD45^+^Collagen I^+^) in Saline, OVA and IL-25 treated lung tissue samples were shown. Proteins detected using respective antibodies are indicated on top. The arrows indicated positive cells with the co-expression of CD45, Collagen I, and IL-17RB proteins in lung tissues. Images are representative of five biological replicates. Bar represents 100 μm. **B** and **C**: Statistical analysis for the percentages of total fibroctyes (CD45^+^Collagen I^+^) and IL-17RB expressing fibrocytes (IL-17RB^+^ CD45^+^Collagen I^+^). Bars represent the mean ± SEM of 5 mice. *p < 0.05, **p < 0.01, ***p < 0.001 by using one-way ANOVA with Tukey post hoc analysis

We previously showed that OVA and IL-25 challenge induces significant inflammatory and fibrotic changes of airway and lung tissues [28–30]. The present results showed that the number of infiltrating CD45^+^Collagen I^+^- and IL-17RB^+^CD45^+^Collagen I^+^-fibrocytes were higher in peribronchial areas and lung parenchyma treated with OVA and IL-25 than in those treated with saline (Fig. 2A-C).

### IL-25 facilitated pro-fibrotic phenotypic changes in BECs partially via the PI3K signal pathway

**Fig. 3:**
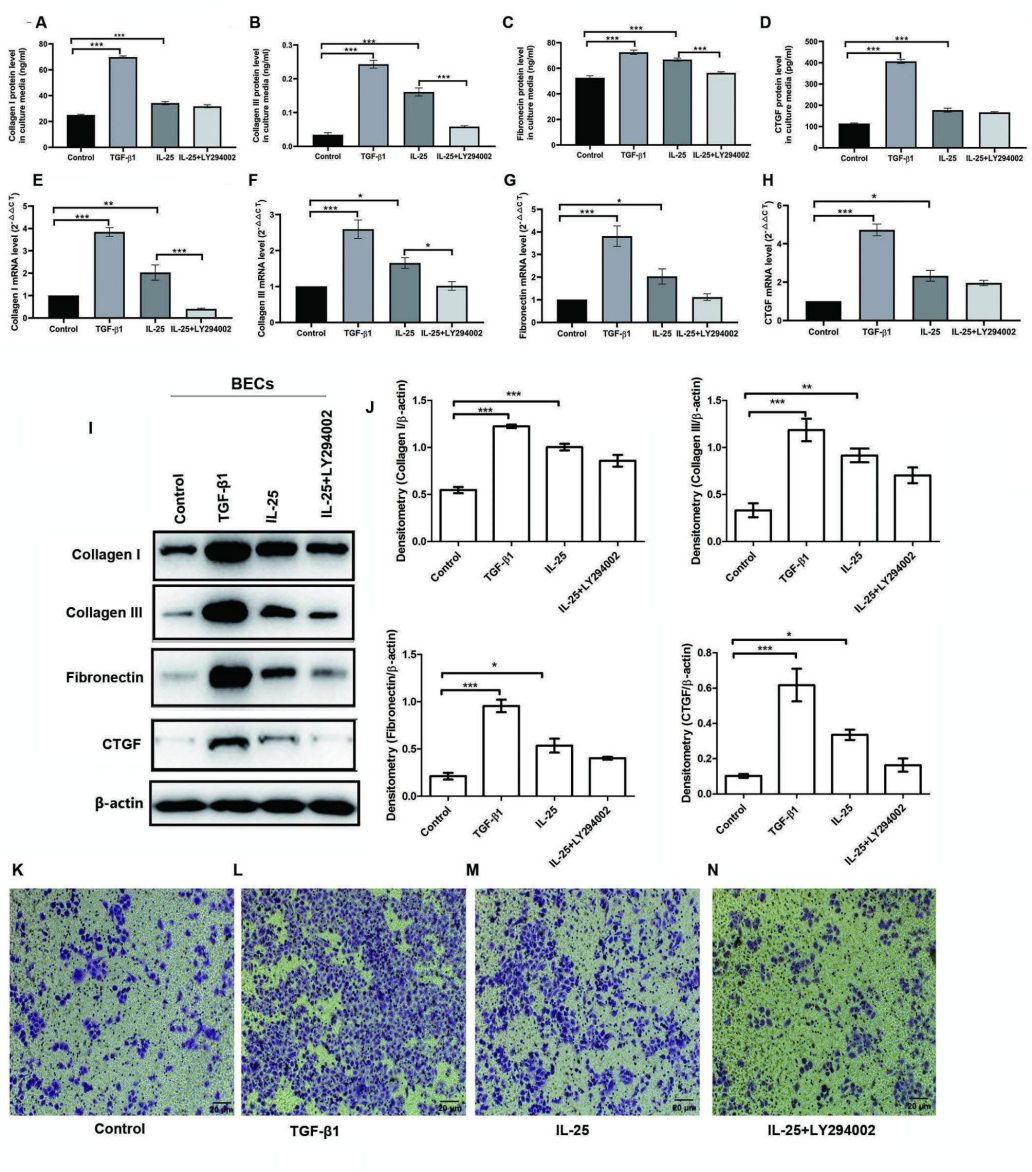
IL-25 promotes pro-fibrotic phenotypic changes in BECs and was partially blocked with LY294002. BECs were stimulated with complete medium alone (control), TGF-β1 (10 ng/ml), IL-25 (10 ng/ml) and IL-25 + LY294002 (15 μM) for 24 h. The extracellular protein levels of Collagen I (**A**), Collagen III (**B**), Fibronectin (**C**), and CTGF (**D**) in BECs were analyzed by using ELISA assays. The levels of Collagen I (**E**), Collagen III (**F**), Fibronectin (**G**), and CTGF (**H**) mRNAs in BECs were analyzed by quantitative real-time PCR. Western blot analysis (I and **J**) was used for determining the intracellular protein levels of Collagen **I**, Collagen III, Fibronectin, and CTGF in BECs. β-actin was used as an internal control. BECs migration (**K**-**N**) were determined by using Transwell assay. Bars represent the mean ± SEM of 3 ~ 4 independent experiment. *p < 0.05, **p < 0.01, ***p < 0.001 by using one-way ANOVA with Tukey post hoc analysis

**Fig. 4:**
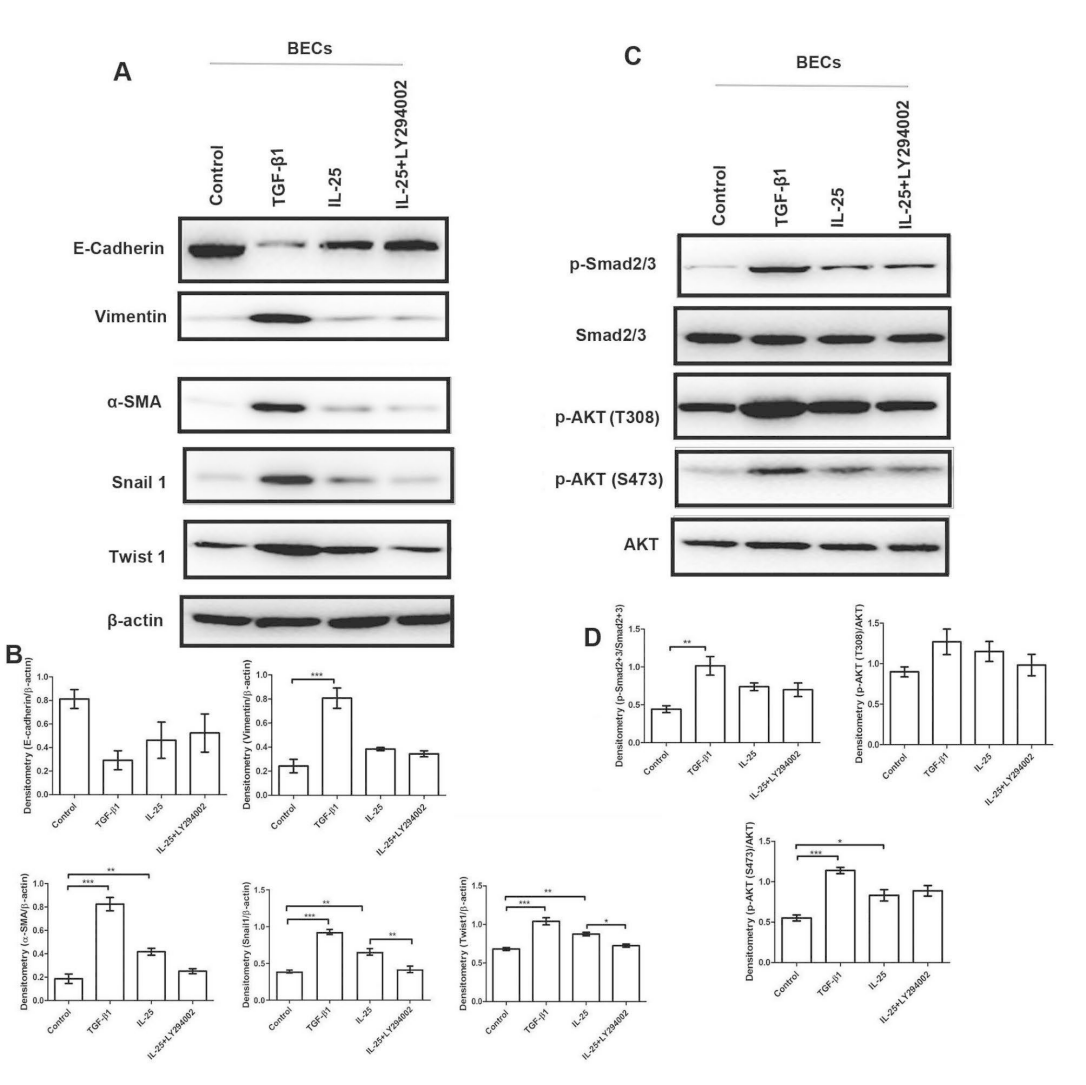
Expression changes of EMT markers in BECs upon IL-25 treatment. BECs were stimulated with complete medium alone (control), TGF-β1 (10 ng/ml), IL-25 (10 ng/ml) and IL-25 + LY294002 (15 μM) for 24 h. **A**: The intracellular protein levels of epithelial marker E-Cadherin, mesenchymal maker vimentin, EMT regulator Snail 1 and Twist 1 in BECs were detected by Western blot analysis; **B**: Statistical data for the relative protein level of E-Cadherin, Vimentin, Snail 1 and Twist 1 to β-actin were shown; **C**: The protein levels of p-Smad2/3, p-AKT (T308) and p-AKT (S473) were detected by Western blot analysis; D: Statistical data for relative protein levels of p-Smad2/3 and p-AKT were shown. Bars represent the mean ± SEM of 4 independent experiment. *p < 0.05, **p < 0.01, ***p < 0.001 by using one-way ANOVA test followed by Turkey multiple-comparison test

IL-25 significantly increased the extracellular and intracellular mRNA and protein levels of Collagen I, Collagen III, Fibronectin, and CTGF (Fig. 3A-J). IL-25 had a similar but weaker effects compared to TGF-β1, a master regulator of EMT and organ fibrosis [36]. The profibrotic effect of IL-25 was partially blocked by LY294002, a specific antagonist of the PI3K signaling pathway (Fig. 3A-J). Furthermore, transwell migration assay showed that IL-25 and TGF-β1 treatment both markedly enhanced the ability of BECs to translocate across the micropores of the inserts, which can be evidently inhibited by LY294002 (Fig. 3K-N). As shown in Fig. 4A-B, IL-25 had a similar but weaker effect with TGF-β1 in decreasing E-cadherin expression and increasing vimentin, α-SMA, snail1, and twist 1 expression in BECs. This suggests that IL-25 promoted an EMT-like changes in BECs. The EMT-like changes in BECs induced by IL-25 were partially blocked by LY294002 (Fig. 4A-B). IL-25 also triggered phosphorylation of Smad2/3 (p-Smad2/3) expression in BECs (Fig. 4C-D).

### IL-25 promoted pro-fibrotic phenotypic changes in CFs partially via PI3K signal pathway

**Fig. 5:**
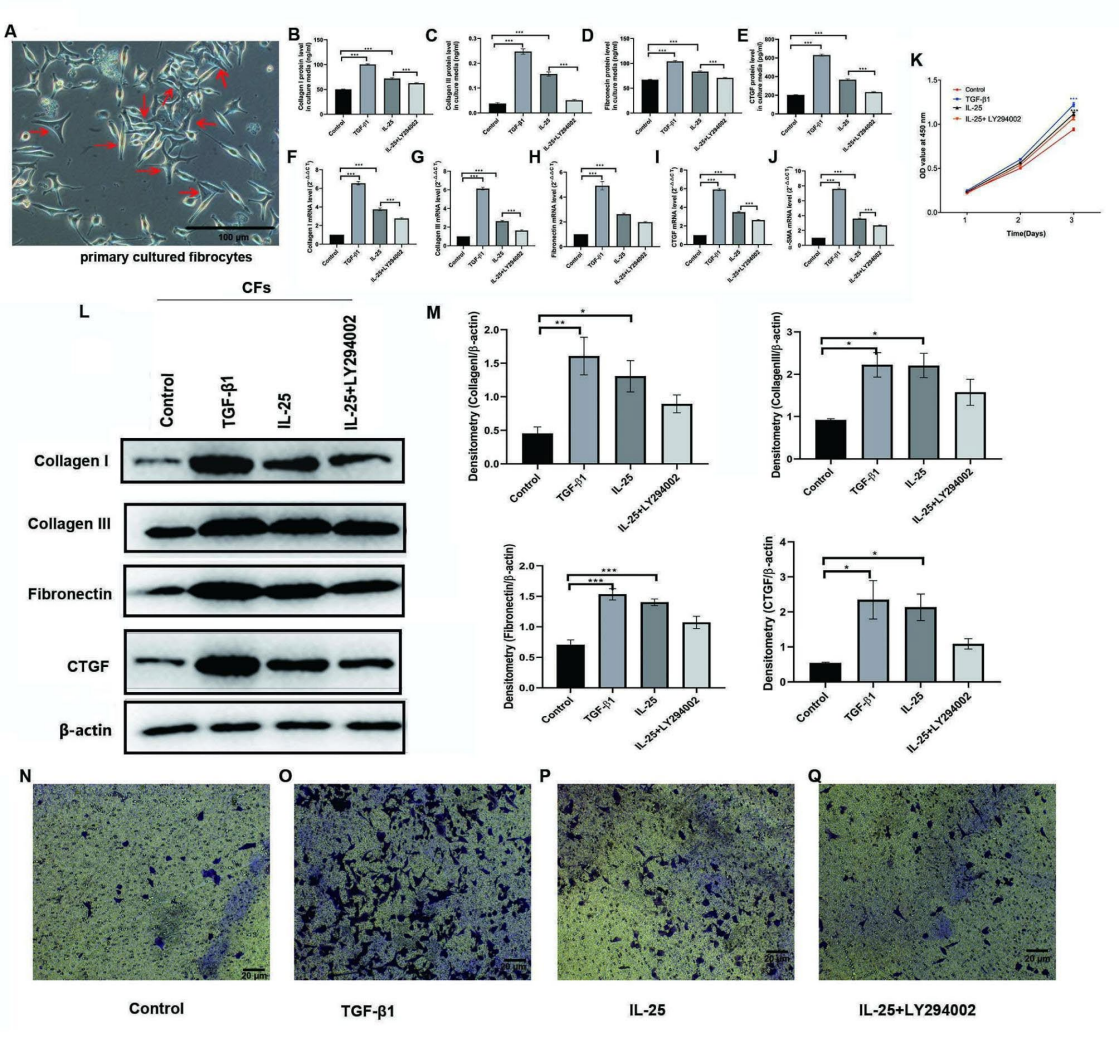
IL-25 promotes pro-fibrotic phenotypic changes in CFs and was partially blocked with LY294002. **A**: Representative image of primary cultured CFs from NANT PBMCs in asthma patients taken with a phase-contrast microscope. Red arrows indicated the typical fibrocytes. Bar represents 100 μm. **B-E**: CFs were stimulated with complete medium alone (control), TGF-β1 (10 ng/ml), IL-25 (10 ng/ml) and IL-25 + LY294002 (15 μM) for 24 h. Collagen I (B), Collagen III (C), Fibronectin (D), and CTGF (E) in culture medium from CFs were analyzed by using ELISA assays. **F-J**: CFs were stimulated with complete medium alone (control), TGF-β1 (10 ng/ml), IL-25 (10 ng/ml) and IL-25 + LY294002 (15 μM) for 24 h. The levels of Collagen I (F), Collagen III (G), Fibronectin (H), CTGF (I) and α-SMA (J) mRNAs in CFs were analyzed by real-time RT-PCR. **K**: CFs were stimulated with complete medium alone (control), TGF-β1 (10 ng/ml), IL-25 (10 ng/ml) and IL-25 + LY294002 (15 μM) for 24, 48, and 72 h. The proliferation potential of differentially treated CFs was determined by using CCK-8 assay. **L** and **M**: CFs were stimulated with complete medium alone (control), TGF-β1 (10 ng/ml), IL-25 (10 ng/ml) and IL-25 + LY294002 (15 μM) for 24 h. The intracellular protein levels of Collagen I, Collagen III, Fibronectin, and CTGF in CFs were detected by using Western blot assay. β-actin was used as an internal control. **N-Q**: CFs were stimulated with complete medium alone (control), TGF-β1 (10 ng/ml), IL-25 (10 ng/ml) and IL-25 + LY294002 (15 μM) for 24 h. The the migration ability of differentially treated CFs was determined and compared by using Transwell chamber assay. Data were analyzed using the one-way ANOVA with Tukey post hoc analysis and displayed as mean ± SEM of 3 ~ 4 separate experiments, *p < 0.05, **p < 0.01, ***p < 0.001

As observed in phase-contrast light microscopy, the CFs were elongated cells with a spindle-shaped morphology (Fig. 5A). IL-25 and TGF-β1 significantly upregulated the extracellular or cellular mRNA or protein levels of Collagen I, Collagen III, Fibronectin, CTGF, and α-SMA in the CFs (Fig. 5B-J L-M). PI3K inhibitor LY294002 markedly or partially blocked the expressions of these molecules induced by IL-25. IL-25, like TGF-β1, facilitated the proliferation of CFs in a time-dependent manner, which became most apparent at 72 h (Fig. 5K). Finally, as shown in Fig. 5N-Q, IL-25 had a similar effect but with a much lower effect than TGF-β1 in promoting the migration of CFs.

### IL-25 mediated the abnormal crosstalk from BECs to CFs and promoted the pro-fibrotic phenotypic changes in co-cultured CFs in a paracrine dependent manner

**Fig. 6:**
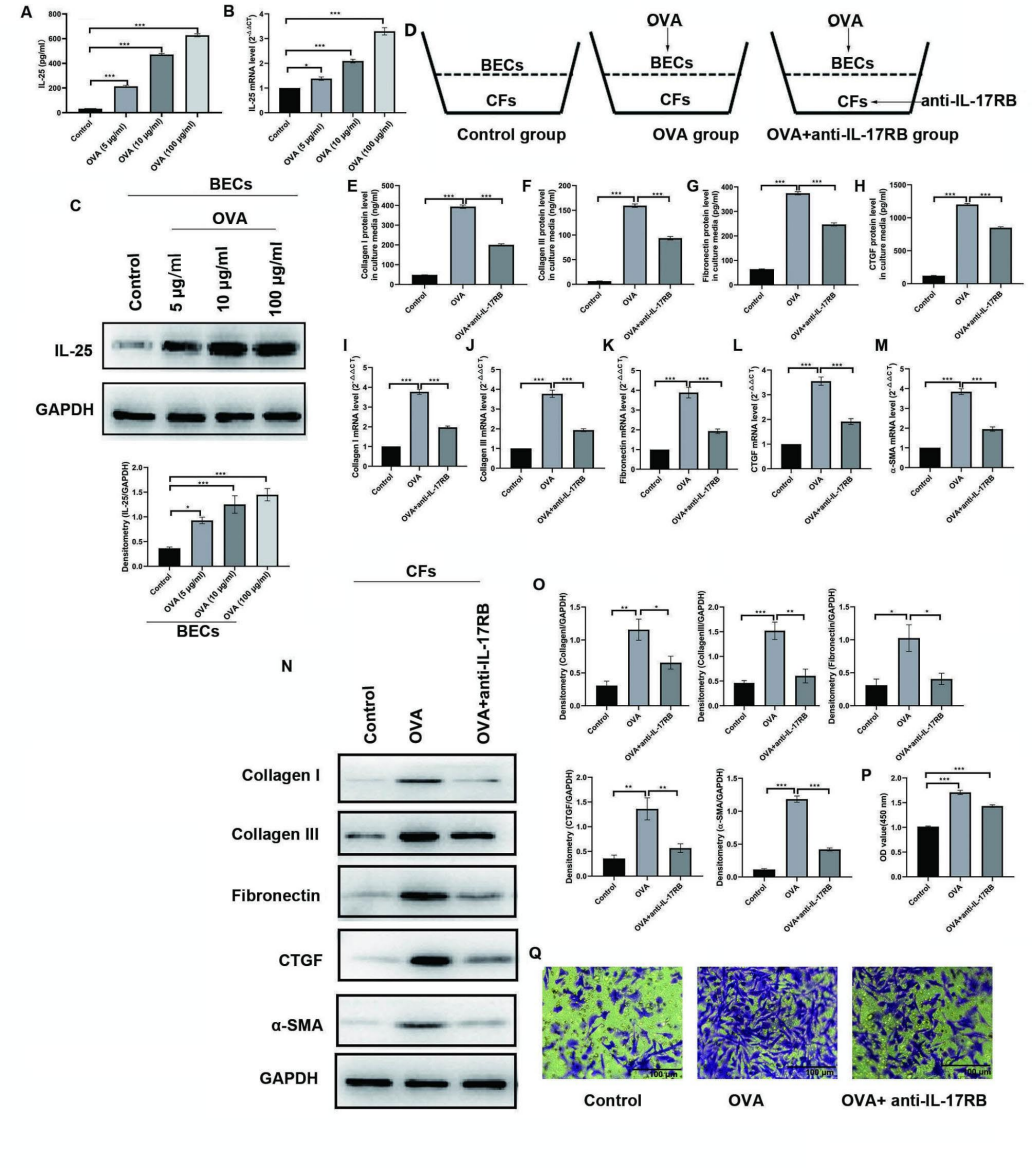
IL-17RB antibody blocked OVA induced pro-fibrotic crosstalk from BECs to CFs. **A-C**: BECs were stimulated with complete medium alone (control) and OVA at various concentrations (i.e., 5, 10 and 100 μg/ml) for 24 h. The mRNA (B), extracellular (A) and intracellular (C) protein levels of IL-25 were detected and compared by using real-time RT PCR, ELISA and Western blot assays, respectively. **D**: Co-culture models of CFs with BECs and grouping: left panel, both BECs and CFs were treated with complete medium alone (control); middle panel, BECs were treated by OVA, while CFs were treated with complete medium alone; right panel, BECs were treated by OVA, while CFs were treated with anti-IL-17RB antibody (1 μg/ml). **E-Q**: CFs were co-cultured with BECs for 24 h as indicated before. Then CFs was harvested for further analysis. **E**-**H**: The protein levels of Collagen I (**E**), Collagen III (**F**), Fibronectin (**G**), CTGF (**H**) in culture medium of co-cultured CFs were detected by using ELSIA assay; **I**-**M**: The levels of Collagen I (**I**), Collagen III (**J**), Fibronectin (**K**), CTGF (**L**) and α-SMA mRNAs (**M**) in co-cultured CFs were analyzed by real-time RT-PCR. **N**: The protein levels of Collagen **I**, Collagen III, Fibronectin, CTGF, and α-SMA in cell lysates of co-cultured CFs were detected by using Western blot assay. **O**: Statistical data were shown for the Western blot assay. GAPDH was used as the loading control for signal normalization and the relative quantification of densitometric unit. **P**: The proliferation ability of co-cultured CFs was determined by using CCK-8 assay. **Q**: The migration ability of co-cultured CFs was determined and compared by using Transwell chamber assay Error bars denote the mean ± SEM of 3 ~ 4 independent experiment. *p < 0.05, **p < 0.01, ***p < 0.001 by using one-way ANOVA test followed by Turkey multiple-comparison test

OVA significantly increased the mRNA and intracellular and extracellular protein levels of IL-25 in BECs (Fig. 6A-C). BECs and CFs were then co-cultured in Transwell® chambers (Fig. 6D). After 24 h culture of BECs in the presence of OVA (10 μg/ml), a significant increase in the gene expression and protein levels of Collagen I, Collagen III, Fibronectin, CTGF, and α-SMA was observed in co-cultured CFs (Fig. 6E-O). In addition, the proliferation and migration of co-cultured CFs were markedly enhanced by OVA-treated BECs (Fig. 6P-Q). Blocking IL-25 significantly attenuated the expression of profibrotic genes and proteins and the potential for proliferation and migration in co-cultured CFs induced by OVA-treated BECs (Fig. 6E-Q).

## Discussion

This study showed that IL-17RB^+^ fibrocytes were increased in the blood circulation of asthmatic patients with FAL and in the lung tissues of chronic murine asthma models. Administration of exogenous IL-25 facilitated an “EMT-like” change in BECs and promoted the pro-fibrotic phenotypic changes of both BECs and primary cultured CFs partially dependent on PI3K-AKT signaling. Endogenous BEC-derived IL-25 was found to potentially mediate pathological crosstalk between BECs and CFs, thereby promoting profibrotic phenotypic changes in CFs. Collectively, we support the potential role for IL-25 in COA by modulating the pro-fibrotic phenotypic changes of BECs and CFs in an autocrine- and paracrine-dependent manner, respectively (Fig. S2).

CFs are bone marrow-derived, circulating mesenchymal progenitor cells that can be recognized by their distinct cellular markers, CD45 and Collagen I [21, 22]. It is well-known that CFs increase in the circulation of patients with asthma and are correlated with asthma severity, chronic airflow obstruction, and acute asthma exacerbation [12–14, 20]. CFs have been shown to respond to various cytokines and chemokines [11, 13, 19, 37, 38]. In our previous study, we showed that the IL-25 receptor, IL-17RB, can be expressed by CFs of patients with asthma [15]. Asthmatic patients with FAL (defined as FEV_1_/FVC < 0.70 [1, 3]) had significantly higher levels of total and IL-17RB ^+^-CFs compared than those without FAL (Fig. 1A-B), similar to previous studies. Because compared with asthma patients without FAL, patients with FAL were male dominant and older (Table S2), there was one possibility that the increased proportion of CFs and IL-25R^+^-CFs was caused by age or gender difference. However, statistical analysis showed that both total CFs and IL-17RB^+^-CFs were comparable between male and female patients, and the proportions of CFs and IL-25R^+^-CFs were not significantly correlated with ages (Data were not shown). This indicated that ages and genders were not the risk factors for higher numbers of CFs. In addition, IL-17RB ^+^-CFs were correlated with FEV_1_/FVC in patients with asthma (Fig. 1D). IL-17RB ^+^-fibrocytes were also increased in the lung sections of chronic allergen- and non-allergen-induced mouse asthma models (Fig. 2). This indicates that IL-17RB ^+^-CFs may be a disease marker for asthma with FAL. Future studies should be conducted using a large asthma cohort to determine whether the percentage or number of IL-17RB^+^-CFs in the circulation can discriminate asthma patients with FAL from those without.

In addition, we believe that the IL-25-(IL-17RB ^+^)-CFs axis is not only a disease biomarker for asthma with FAL but also an important contributor to airway fibrosis and remodeling. Results showed that exogenous IL-25 challenge can induce a remarkable pro-fibrotic phenotypic change in CFs (Fig. 5). In line with our study, Bianchetti et al. [39] showed that IL-33 induces the migration and proliferation of CFs from patients with asthma. Taken together, our results and those of previous studies support the notion that circulation-derived fibrocytes show high phenotypic plasticity in response to the surrounding milieu.

More importantly, we confirmed that IL-25 mediates pathological profibrotic communication from BECs to CFs. In the BECs-CFs co-culture system, OVA-stimulated BECs initiated aberrant pro-fibrotic crosstalk with co-cultured CFs, which could be significantly blocked using an anti-IL-17RB antibody (Fig. 6). IL-25 binds to its receptor, which is composed of the heterodimer complex of IL-17 receptor A (IL-17RA) and IL-17 receptor B (IL-17RB) for signal transduction [40]. However, the biological effects of IL-25 are reported to be mediated mainly through IL-17RB because IL-25 has a higher affinity for IL-17RB than for IL-17RA, and it activates signaling pathways through IL-17RB [41].

Moreover, we also confirmed that IL-25 contributes airway fibrotic remodeling by mediating pro-fibrotic and “EMT”-like phenotypic changes of BECs (Figs. 3–4). Type 2 EMT is known as a cellular program that is known to be crucial for physiologic wound healing and pathological organ fibrotic remodeling [42]. Previous studies have shown that exposure to growth factors, inflammatory mediators, and allergens can induce the down-regulation of epithelial cell-cell adhesions and promote mesenchymal gene expression both in vitro and in vivo in asthmatic airways, demonstrating that the airway epithelium may contribute to airway remodeling via EMT in asthma [43, 44]. In the present study, we presented novel evidence of IL-25 contributing to airway fibrotic remodeling by facilitating EMT”-like pro-fibrotic phenotypic changes in BECs in an autocrine-dependent manner.

Finally, we preliminarily examined the post-receptor signal transduction pathway underlying the modulatory effects of IL-25 on pro-fibrotic phenotypic changes in BECs and CFs. PI3K-specific inhibitor LY294002 has been shown to significantly block IL-25-induced basic fibroblast growth factor (bFGF) expression in human vascular endothelial cells (HUVECs) [41]. Here, we showed that the profibrotic effect of IL-25 on BECs and CFs could be partially blocked using LY294002 (Figs. 3, 4 and 5). However, the inhibitory effect of LY294002 was not very significant. This could be attributed to several factors. First, the concentration of LY294002 used in our experiment was 15 μmol/L, which may not be enough to completely block the PI3K pathway. This was verified by the fact that p-Akt (T308 and S473) was only weakly reduced by LY294002 (Fig. 4C). Second, other post-receptor pathways may also be involved in the pro-fibrotic effects of IL-25, as IL-25 stimulation also promoted the expression of p-Smad2/3 in BECs (Fig. 4C). Smad2/3 is the canonical intracellular signaling pathway underlying TGF-β that has been implicated in the pathogenesis of organ fibrosis [45]. Our results indicate that the post-receptor signal transduction pathways of IL-25 may be diverse and should be further studied.

Our study has several limitations. First, we suggest that IL-25- and IL-17RB^+^-CFs may serve as specific disease markers in patients with asthma and FAL. However, this requires verification in a large cohort study. Second, we showed that both OVA and IL-25 administration induced a significant accumulation of fibrocytes in asthmatic airways. However, we did not show whether IL-25 blockage could reduce the OVA-induced airway accumulation of fibrocytes, thereby reducing airway remodeling. Despite these limitations, our study elucidated the role and mechanism of IL-25 in asthmatic airway remodeling.

## Conclusions

We suggest that IL-25 contributes to asthmatic airway remodeling and fibrosis by directly acting on BECs and CFs in an autocrine and paracrine dependent manner, respectively. IL-25 blocking therapy may serve the potential paradigm for the treatment of chronic obstructive asthma.

### Electronic supplementary material

Below is the link to the electronic supplementary material.

Supplementary Material 1

## Data Availability

The datasets used and/or analysed during the current study are available from the corresponding author on reasonable request.

## List of abbreviations

BECs: Bronchial epithelial cells
CFs: Circulating fibrocytes
COA: Chronic obstructive asthma
CTGF: Connective tissue growth factor
ECM: Extracellular matrix
EMT: Epithelial-mesenchymal transition
FAL: Fixed airflow limitation
FAO: Fixed airway obstruction
FeNO: Fraction of exhaled NO
FEV_1_/FVC: Forced expiratory volume in 1 s/forced vital capacity
ICS: Inhaled corticosteroids
IL-25: Interleukin 25
OVA: Ovalbumin
TSLP: Thymic stromal lymphopoietin
