# Supplementary material for: IL-25 induces airway remodeling in asthma by orchestrating the phenotypic changes of epithelial cell and fibrocyte
Source: Respir Res. 2023 Aug 27;24:212. doi: 10.1186/s12931-023-02509-z (PMC10463650; doi:10.1186/s12931-023-02509-z)
Supplement: Supplementary file 1 — Supplementary Material 1 [file 12931_2023_2509_MOESM1_ESM.docx]

**SUPPLEMENTARY INFORMATION**

**Title：**IL-25 leads to airway remodeling in asthma by orchestrating the phenotypic changes of epithelial cell and fibrocyte

**Authors:** Xiujuan Yao^1^, Qinglin Chen^1^, Xiangdong Wang^2,3^, Xiaofang Liu^1^*, Luo Zhang^2,3^*

**Affiliations:** **^1^** Department of Respiratory and Critical Care Medicine, Beijing Tongren Hospital, Capital Medical University, Beijing, China; **^2^** Department of Otolaryngology Head and Neck Surgery, Beijing Tongren Hospital, Capital Medical University, Beijing, China; **^3^** Key Laboratory of Otolaryngology Head and Neck Surgery of Ministry of Education of China, Beijing Institute of Otolaryngology, Beijing, China

***Corresponding authors:** **(1)** **Luo Zhang**, MD, PhD, Key Laboratory of Otolaryngology Head and Neck Surgery of Ministry of Education of China, Beijing Institute of Otolaryngology, No. 17, Hougou Hutong, Dongcheng District, Beijing 100005, China. Tel.: +86-10-65141136, Fax: +86-10-85115988, Email address: dr.luozhang@139.com; **(2) Xiaofang Liu**, MD, Department of Respiratory and Critical Care Medicine, Beijing Tongren Hospital, Capital Medical University. No.2, Xinanhuan Road, Yizhuang District, Beijing 100000, China. Tel.: +86-10-58266058, Email address: xfliutrhos@163.com.

**Materials and Methods**

**Flow cytometry**

Briefly, peripheral blood mononuclear cells (PBMCs) were separated from 20 ml of whole blood from asthma patients by using Lymphoprep™ (Axis-Shield, Oslo, Norway) density gradient centrifugation. Non-adherent, non-T (NANT) cells from PBMCs were then harvested by adherent culture for 2 h followed by T cell depletion with magnetic beads coated with anti-CD3 mAb (5 µl, Miltenyi Biotech, Paris, France). NANT-PBMCs were firstly stained with fixable viability stain 440UV (Becton, Dickinson and Company, Franklin Lakes, NJ, USA) for cell viability assessment. Then, the cells were first stained with Brilliant Violet 421™-anti-human CD3 (5 µl, BioLegend, San Diego, CA, USA) to remove CD3^+^ PBMCs. The cells were then stained with BV480-anti-human CD45 (5 µl, Becton, Dickinson and Company) and PE-anti-human IL-17RB (8 µl, R&D Systems, Minneapolis, MN, USA) monoclonal antibodies. After permeabilization using FACS permeabilizing solution (Becton Dickinson, Sparks, MD, USA), intracellular staining of collagen was performed with FITC-anti-human Collagen I monoclonal antibody (5 µl, Milli-Mark™, Millipore Corporaion, Burlington, MA, USA). Additionally, the fluorescence minus one (FMO) controls were applied for each marker, in which each fluorophore in turn was excluded. The fluorescently-labelled cells was finally analyzed with a FACSymphony^TM^ A5 flow cytometer (Becton, Dickinson and Company). Flow Jo^®^ version 7.6 software (Tree Star, Inc., Ashland, OR, USA) was used for data analysis. Positive or negative gates for each marker was set by using FMO controls.

**Murine model of asthma**

Briefly, female BALB/c wild-type mice (8-10 weeks old, Vital River Laboratories, Beijing, China) were allocated into 3 groups, including OVA challenged group (a “classical” allergen-induced asthma model), IL-25 challenged group (a non-allergen induced asthma model), and saline group served as a negative control. Mice in OVA challenged group were first sensitized by intraperitoneal injection of OVA (Sigma-Aldrich, Beijing, China, 100 μg emulsified in Al[OH]_3_) at days 0 and 12. Then 50 μg of OVA in 50 μL saline were further administered daily by nasal instillation from days 18 to 23. Mice challenged with IL-25 received daily nasal instillation with recombinant mouse IL-25 (mIL-25, R&D Systems, 2 μg in 50 uL saline) from days 18 to 23. Subsequently, mice were further challenged intranasally either with OVA or with IL-25 every 2 days for a further 30 days. Mice in saline group was intraperitoneally injected with the same amount of Al[OH]_3_ on days 0 and 12, then nasally administered with saline at time points corresponding to those in OVA and IL-25 challenged groups. Finally, mice were sacrificed by euthanasia at day 55 for lung tissue preparation.

**Multiplex immunohistochemistry and multispectral image analysis**

Anti-CD45, Collagen I and IL-17RB primary antibodies were sequentially applied to a single slide. Briefly, slide was first deparaffinized in xylene, rehydrated in ethanol and undergone antigen retrieval in citrate buffer (pH=6.0) using microwave treatment. After blocked with bovine serum albumin (BSA), rabbit anti-CD45 mAb (1:200, Cell Signaling Technology [CST], Danvers, MA, USA) were incubated for 30 min in a humidified chamber at room temperature (RT), followed by horseradish peroxidase (HRP)-conjugated secondary antibody incubation (Panovue, Beijing, China) at RT for 10 min. Visualization of CD45 was accomplished by using fluorescein PPD650 after the slide was microwave heated in antigen retrieval solution. Accordingly, in a serial fashion, the same slide was subsequently incubated with rabbit polyclonal antibody to Collagen I (1:100, Abcam, Shanghai, China) and goat polyclonal antibody to IL-17RB (1:100, Thermo Fisher Scientific, Shanghai, China). After secondary antibody incubation, fluorescein PPD520 and PPD620 were used for the visualization of Collagen I and IL-17RB, respectively. Finally, nuclei were subsequently stained with 4'-6'-diamidino-2-phenylindole (DAPI, Sigma-Aldrich, Shanghai, China) for 5 min and enclosed in ProLong Diamond Antifade Mountant (Thermo Fisher Scientific). The slides were scanned by using the Mantra System (PerkinElmer, Waltham, MA, USA) and the multispectral images obtained were unmixed using spectral libraries that were previously built from images stained for each fluorophore, using the inForm image analysis software (PerkinElmer, Waltham, Massachusetts, USA).

**Primary circulating fibrocytes (CFs) culture**

PBMCs was collected from peripheral blood in 6 independent asthma patients with FAL under ethical approval as described before. PBMCs was washed twice with 5 ml of PBS and harvested by centrifugation at 500 g for 10 min. The cells were then re-suspended in dulbecco's modified eagle medium (DMEM) (Thermo Fisher Scientific) containing 20% fetal bovine serum (FBS, Thermo Fisher Scientific), penicillin (100 U/ml) and streptomycin (100 μg/ml, all from Thermo Fisher Scientific). After that, the cells were seeded into 6-well plates coated with 10 μg/mL fibronectin (Sigma-Aldrich) at a density of 1×10^6^/ml and cultured in a humidified incubator at 37˚C in 5% CO_2_. After 2 days, the non-adherent cells were aspirated off, and the remaining adherent cells were cultivated for additional 10-14 days prior to experiment. The culture media were changed every 3 days and cells received passage culture when they had reached confluence. The morphological features of CFs were observed under an inverted phase contrast microscope.

**Real time RT PCR**

In brief, total RNA in the cellular homogenates was collected using a RNAprep pure Cell/Bacteria Kit (TIANGEN, Beijing, China) according to the manufacturer's protocol. cDNA was then synthesized by RT-PCR with a FastKing One Step RT-PCR Kit (TIANGEN). Quantitative real-time PCR was subsequently performed on Applied Biosystems™ 7500 Real-Time instruments (Thermo Fisher Scientific) with a standard protocol by using a SuperReal PreMix Plus Kit (SYBR Green, TIANGEN). The relative gene expression was normalized to GAPDH gene expression and was quantified using the 2^−ΔΔCT^ method.

**Western blot**

In brief, total proteins from cultured cells were extracted in radioimmunoprecipitation assay (RIPA; Pierce, Rockford, IL, USA) buffer supplemented with Proteinase and Phosphatase Inhibitor Cocktails (Roche, Basel, Switzerland). Protein concentrations were determined by using the bicinchoninic acid (BCA) assay kit (Pierce). Protein samples (30 µg/lane) were separated with 8%-12% SDS-PAGE and transferred onto nitrocellulose (NC) filter membranes (Millipore). After blocking with 5% bovine serum albumin (BSA) in TBS-0.1% Tween-20 solution, NC membranes were incubated with the indicated primary antibodies overnight at 4°C according to the manufacturer's instructions, including mouse/rabbit anti-IL-25 (1:1000), anti-Collagen I (1:1000), anti-Collagen III (1:5000), anti-Fibronectin (1:1000), anti-CTGF (1:500), anti-α-SMA (0.1µg/ml), anti-E-Cadherin (1:1000), anti-Vimentin (1:1000), anti-Snail1 (10 µg/ml), andti-Twist1(1 µg/ml), anti-p-Smad2/3 (1:1000), anti-Smad2/3 (1:1000)，anti-p-AKT (T308, 1/500)，anti-p-AKT (S473, 1/5000), anti-GAPDH (1/500) and anti-β-actin (1/5000) antibodies (all from Abcam). After that, the membranes were incubated with a horseradish peroxidase-conjugated polyclonal secondary antibody (Abcam) for 1 h at room temperature. Protein bands were visualized with enhanced chemiluminescence (ECL) solution and a ChemiDoc XRS+ system (Bio-Rad Laboratories, Inc., Hercules, CA, USA). The densities of the bands were finally analyzed with Image J software v1.51j8 (National Institutes of Health [NIH], Bethesda, MD, USA).

**Table S1. PCR primer sequences**

| **Genes** | **Sequences** |
| --- | --- |
| **Collagen I** |  |
| **Forward** | GGGCAAGACAGTGATTGAATA |
| **Reverse** | ACGTCGAAGCCGAATTCCT |
| **Collagen III** |  |
| **Forward** | GAACCAGTGGACATCCAGGT |
| **Reverse** | GGAGCCCTCAGATCCTCTTT |
| **Fibronectin** |  |
| **Forward** | ACCAGCAGAGGCATAAGGTT |
| **Reverse** | GGGTCAAAGCACGAGTCATC |
| **CTGF** |  |
| **Forward** | CGGCGAGGTCATGAAGAAGA |
| **Reverse** | TCATGCCATGTCTCCGTACA |
| **α-SMA** |  |
| **Forward** | TAGCACCCAGCACCATGAAG |
| **Reverse** | CTGCTGGAAGGTGGACAGAG |
| **IL-25** |  |
| **Forward** | TGCTCTACCACAACCAGACT |
| **Reverse** | AAGGAAACACGGTACAGCCT |
| **GAPDH** |  |
| **Forward** | ACCCAGAAGACTGTGGATGG |
| **Reverse** | TCAGCTCAGGGATGACCTTG |

**Note:** All sequences are shown in the 5' to 3' orientation**.**

**Abbreviation:** CTGF, connective tissue growth factor; GAPDH, glyceraldehyde-3-phosphate dehydrogenase; α-SMA, smooth muscle actin α.

**Table S2 Clinical characteristics of study populations**

| **Parameters** | **asthma without FAL** | **asthma with FAL** | ***P* value** |
| --- | --- | --- | --- |
| Numbers | 43 | 37 |  |
| Age(y) | 53.28±2.45 | 67.86±1.81 | <0.0001 |
| Sex (male/female) | 17/26 | 25/12 | 0.0147 |
| Lung function parameters |  |  |  |
| FEV_1_ (L) | 2.52±0.10 | 1.45±0.08 | <0.0001 |
| FEV_1_(%predicted) | 94.73±2.61 | 60.75±2.77 | <0.0001 |
| FVC (L) | 3.18±0.10 | 2.50±0.17 | <0.0001 |
| FEV_1_/FVC (%) | 79.01±1.23 | 56.89±1.56 | <0.0001 |
| Medications | |  |  |
| ICS+LABA (yes/no, n) 37/6 | | 35/2 | 0.2752 |
| Oral corticosteroids (yes/no, n) | 5/38 | 8/29 | 0.2443 |
| Serum Ig E level(IU/ml) | 483.6±103.6 | 426.2±185.2 | 0.4260 |
| FeNO level(ppb) | 88.29±20.25 | 30.90±5.55 | 0.0965 |
| Eosinophil count (×10^9^/L) | 0.47±0.12 | 0.39±0.05 | 0.1847 |

Data were shown as means±SEM; Indicated *P* values between the 2 different groups were calculated with2-sided independent *t* test for parametric variables and Mann-Whitney *U* test for nonparametric variables. For comparison of proportions, the Fisher exact test was used.

Abbreviations: FAL, fixed airflow limitation; FeNO, fractional exhaled nitric oxide; FEV_1_, forced expiratory volume in 1 second; FVC, forced vital capacity; ICS (inhaled corticosteroids); LABA, long-acting bronchodilator.


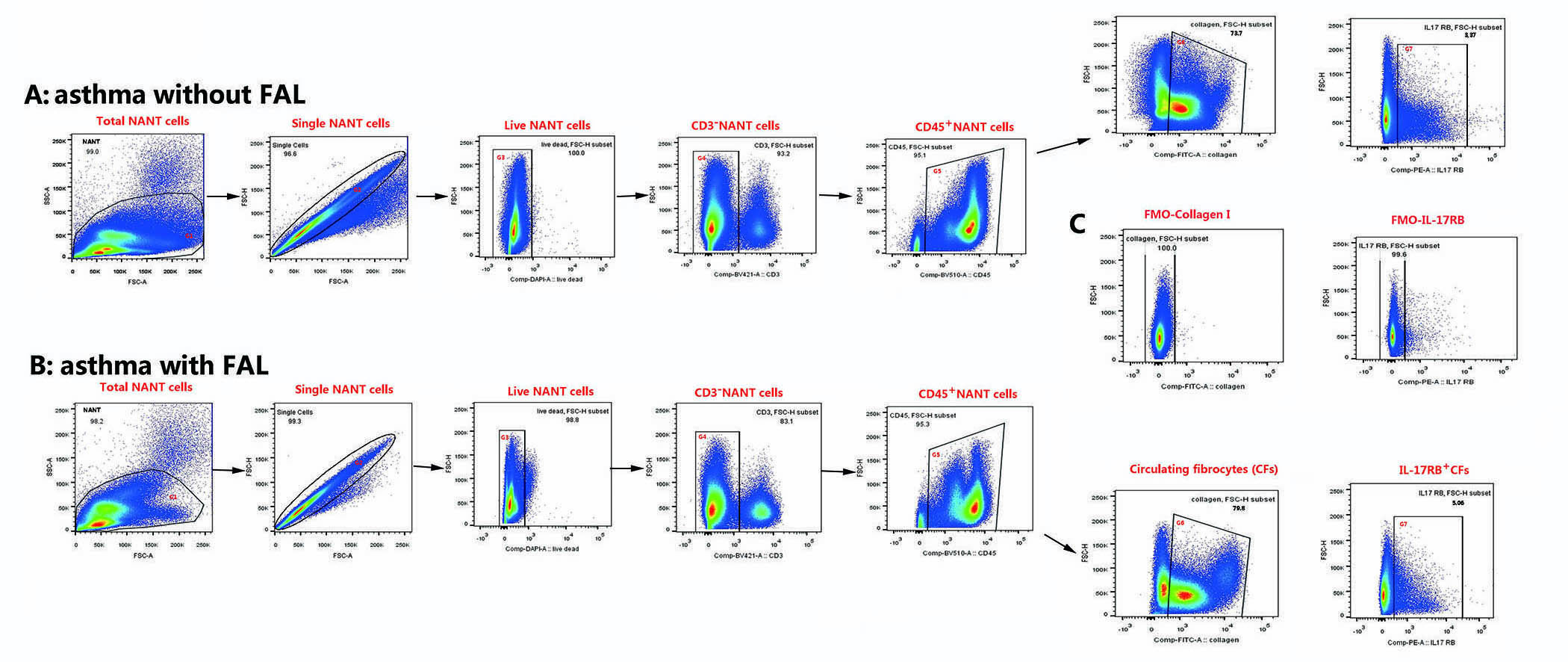


**Fig.S1** **Representative dot plots of flow cytometry for total and IL-25R^+^ circulating fibrocytes (CFs)**

CFs identification and quantification from asthma patient without (A) and with (B) fixed airflow limitation (FAL) were shown.

**A and B:** G1: Total NANT PBMCs were selected with FSC and SSC; G2: Single NANT PBMCs defined by using FSC-H and FSC-A; G3: Live NANT PBMCs determined by using fixable viability stain 440UV; G4: CD3^-^NANT PBMCs determined by using Brilliant Violet 421™-anti-human CD3 antibody; G5: CD45^+^NANT PBMCs determined by using BV480-anti-human CD45 antibody; G6: CFs were defined as CD45^+^Collagen I^+^ NANT PBMCs; G7: IL-25R^+^-CFs were defined as IL-17RB^+^ CD45^+^Collagen I^+^ NANT PBMCs.

**C:** FMO control for Collagen I (left panel) and IL-17RB (right panel).


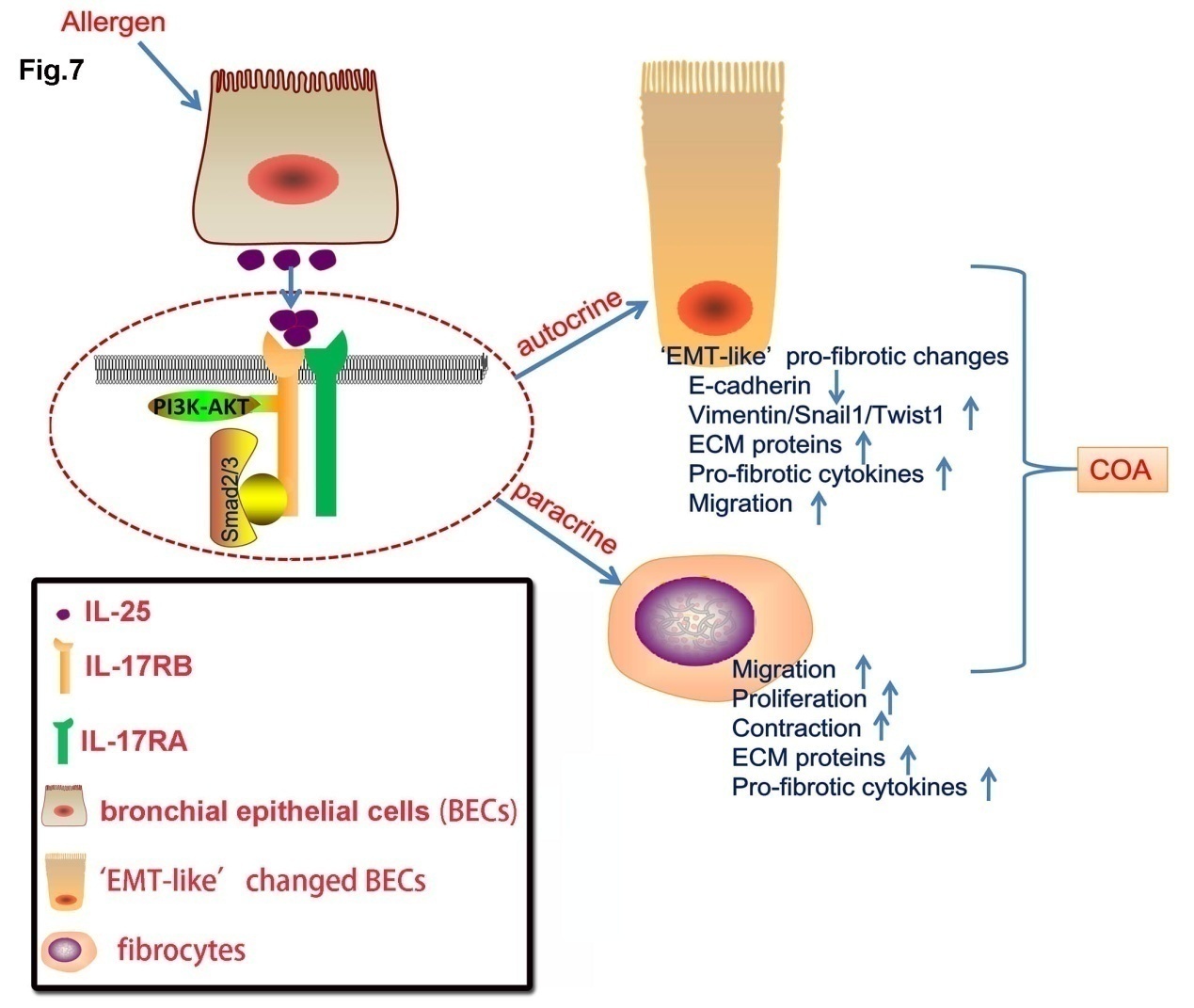


**Figure S2 Schematic of the mechanisms underlying the formation of COA induced by IL-25**

Allergen challenged bronchial epithelial cells (BECs) produced excessive IL-25. IL-25 promoted airway remodeling and the formation of chronic obstructive asthma (COA) in two ways: (1) Autocrine pathway. BECs-derived IL-25 can bind IL-17RA/IL-17RB expressed by BECs and induce a pro-fibrotic 'epithelial-mesenchymal transtion (EMT)'-like phenotypic changes of BECs. This process was characterized by decreased expression of epithelial marker E-cadherin, increased expressions of mesenchymal marker Vimentin, EMT modulators Snail1, Twist1. extracellular matrix (ECM) proteins (including Collagen I, Collagen III, Fibronectin) and pro-fibrotic cytokines (e.g., connective tissue growth factor [CTGF]). The migration of BECs was also enhanced by IL-25. (2) Paracrine pathway. BECs-derived IL-25 can also bind IL-17RA/IL-17RB expressed by fibrocytes derived from the circulation. Then, the injury signal from BECs was transmitted to fibrocytes via IL-25 and induced the pro-fibrotic phenotypic changes of fibrocytes, including enhanced ability of proliferation, migration, contraction, ECM proteins and pro-fibrotic cytokines production. Post-receptor signal pathways of IL-25 may be PI3K-AKT and Smad2/3 dependent.


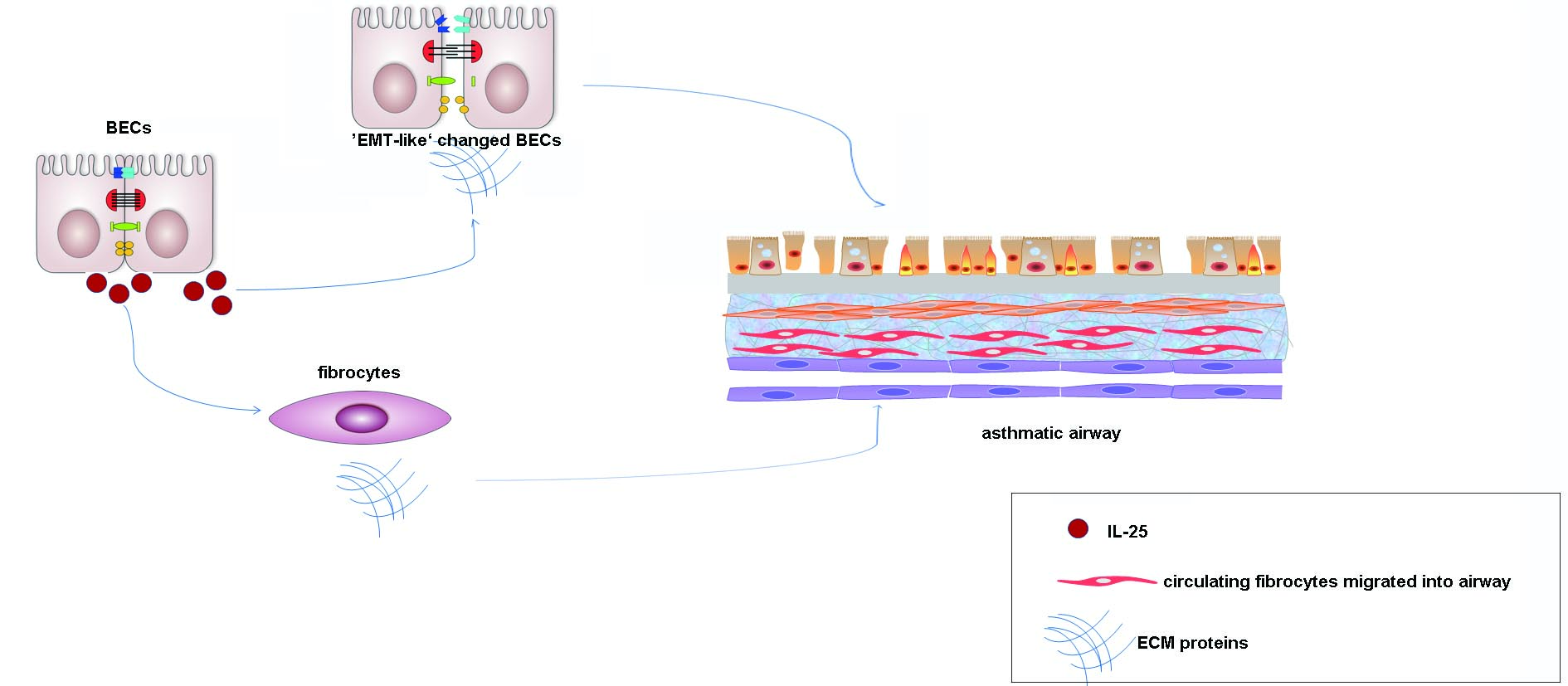


**Graphic abstract**

**The profibrotic role and mechanism of IL-25 in asthma**

1. IL-25 induced an 'EMT-like' and pro-fibrotic phenotypic change of BECs in an autocrine manner;

2. IL-25 induced a pro-fibrotic phenotypic change of fibrocytes in a paracrine manner;

3. IL-25 contributes to asthmatic airway fibrosis and remodeling by modulating the crosstalk from BECs to BECs and to fibrocytes and their phenotypic changes.

Abbreviations: BECs, bronchial epithelial cells; ECM, extracellular matrix; EMT, epithelial-mesenchymal transition; IL, interleukin.
